# Interplay of *GBA1* with lysosomal dysfunction and inflammation in Parkinson’s disease

**DOI:** 10.4103/NRR.NRR-D-25-01082

**Published:** 2025-12-30

**Authors:** Ruochen Wang, Taku Hatano, Nobutaka Hattori, Davide Cossu

**Affiliations:** 1 Department of Neurology, Juntendo University, Tokyo, Japan; 2 Biomedical Research Core Facilities, Juntendo University, Tokyo, Japan; 3 Department of Biomedical Sciences, Division of Microbiology and Virology, University of Sassari, Sassari, Italy

**Keywords:** autophagy–lysosome pathway, glucocerebrosidase (*GBA1*), lysosomal dysfunction, microbiome–gut–brain axis, mitochondrial, neurodegeneration, neuroinflammation, Parkinson’s disease, precision medicine, α-synuclein

## Abstract

Mutations in the glucocerebrosidase (*GBA1*) gene, encoding the lysosomal enzyme glucocerebrosidase, represent the most significant genetic risk factor for Parkinson’s disease. These variants define a distinct clinical subtype characterized by earlier onset, accelerated motor decline, and pronounced cognitive impairment. This review synthesizes current insights into the molecular mechanisms linking *GBA1* dysfunction to lysosomal failure, α-synuclein aggregation, and neuroinflammation. Pathogenic alleles such as N370S and L444P disrupt sphingolipid metabolism, resulting in toxic accumulations of glucosylceramide and glucosylsphingosine, endoplasmic reticulum stress, and impaired clearance of misfolded proteins. This initiates a self-reinforcing cycle in which glucocerebrosidase deficiency promotes α-synuclein aggregation, which subsequently impairs glucocerebrosidase trafficking. We explore the convergence of *GBA1* mutations on the lysosomal–mitochondrial–autophagy axis, where impaired autophagic flux and disrupted organelle crosstalk amplify oxidative stress and activate the NLR family pyrin domain containing 3 inflammasome. The contribution of microglia, astrocytes, and oligodendrocytes to the neuroinflammatory cascade is eamined, along with the emerging influence of the microbiome-gut-brain axis in disease progression. Finally, we evaluate emerging therapeutic strategies, including pharmacological chaperones, NLRP3 inhibitors, adeno-associated virus-based gene therapy, and microbiome modulation, highlighting both promises and translational challenges such as blood–brain barrier penetration and mutation-specific efficacy. We conclude by advocating for precision medicine approaches, supported by robust biomarker development and advanced disease models, to guide tailored interventions for this aggressive Parkinson’s disease subtype.

## Introduction

Parkinson’s disease (PD) is the second most common neurodegenerative disorder worldwide, affecting over 8.51 million individuals in 2021 (GBD 2021 Causes of Death Collaborators, 2024). While the majority of cases are sporadic, a minority are linked to genetic mutations, notably in glucocerebrosidase 1 (*GBA1*), which has emerged as the most prevalent genetic risk factor for PD (Blauwendraat et al., 2020; Bloem et al., 2021; Pachchek et al., 2023; Cook et al., 2024; Yoon et al., 2024), account for 5%–20% of cases across diverse populations, with significant ethnic variability in mutation spectrum and risk (Vieira et al., 2024; Koç Yekedüz et al., 2025). The *GBA1* encodes the lysosomal enzyme glucocerebrosidase (GCase), which is essential for the degradation of glicolipids (Granek et al., 2023; Klein and Outeiro, 2023).

Pathogenic variants such as *N370S* and *L444P* disrupt GCase structure and function, leading to impaired enzymatic activity and accumulation of glycosphingolipids including glucosylceramide (GlcCer) and glucosylsphingosine (GlcSph) (Clemente et al., 2020; Fregno et al., 2025). This lipid disturbance compromises lysosomal degradation, promotes endoplasmic reticulum stress, and reduces clearance of pathological α-synuclein (α-syn) aggregates (Granek et al., 2023; Menozzi et al., 2023). Critically, a toxic feedback loop is established: GCase deficiency facilitates α-syn aggregation, and α-syn oligomers further inhibit GCase trafficking, thereby exacerbating proteostatic collapse (Cui et al., 2022; Wessler and Meisner-Kober, 2025).

The pathogenic mechanisms extend beyond cell-autonomous pathways. *GBA1* dysfunction activates non-cell-autonomous processes involving neuroinflammation, such as microglial NLR family pyrin domain containing 3 (NLRP3) inflammasome signaling, astrocytic reactivity, impaired oligodendrocyte precursor differentiation, and disrupted gut–brain axis communication (Bo et al., 2022; Cossu et al., 2023; Huang et al., 2023; Gregorio et al., 2024). These mechanisms collectively contribute to nigrostriatal degeneration and disease progression.

This review synthesizes recent advances in understanding the molecular pathways linking *GBA1* mutations to lysosomal failure, α-syn pathology, and neuroimmune activation in PD. We also evaluate emerging therapeutic strategies, including pharmacological chaperones, NLRP3 inhibitors, and gene therapy, while addressing ongoing challenges such as blood-brain barrier penetration, mutation-specific efficacy, and the translation of preclinical findings into clinical applications (Höglinger et al., 2022; Smith et al., 2023; Haque et al., 2024). We emphasize the need for biomarker-driven, precision medicine approaches to improve the stratification and treatment of *GBA1*-PD patients.

## Search Strategy

**Figure 1:**
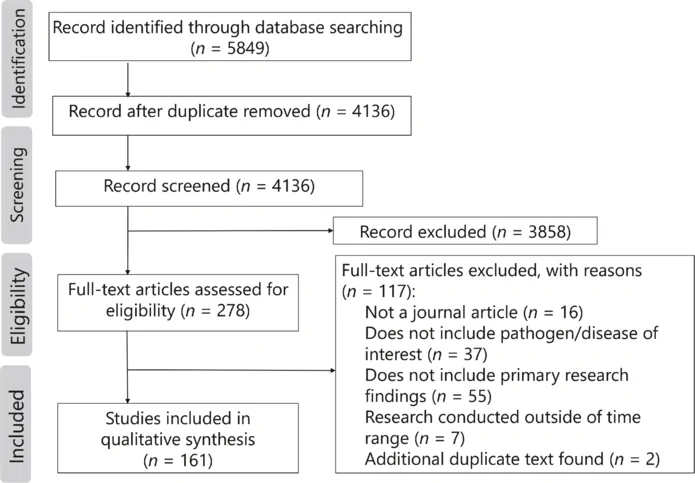
Flow chart of data retrieval and inclusion and exclusion strategies.

A comprehensive search strategy across major electronic databases, including PubMed/MEDLINE, Web of Science, Embase, and the Cochrane Library (inception to June 2025), prioritizing 2020–2025 publications was used in this review. The search utilized core terms such as “*GBA1*,” “glucocerebrosidase,” “Parkinson’s disease,” “lysosomal dysfunction,” “alpha-synuclein,” and “neuroinflammation,” supplemented with pathway-specific keywords. Inclusion criteria encompassed: (1) studies investigating *GBA1*-associated mechanisms (lysosomal impairment, α-syn aggregation, neuroinflammation) or interventions (chaperones, gene therapy); (2) experimental models (cellular, animal, induced pluripotent stem cell [iPSC]-organoids) or human cohorts; (3) quantitative outcomes (GCase activity, cytokine levels, clinical progression). Exclusion criteria comprised non-English publications, uncontrolled case reports, studies published in low-impact journals (JCR Q3/Q4 without raw data), and non-mechanistic applied research. The screening process was conducted in a three-phase process: initial title/abstract triage, full-text evaluation for methodological rigor, and standardized data extraction with cross-model validation of contradictory evidence. The study selection process is summarized in a PRISMA-compliant flowchart (**Figure 1**).

## *GBA1*: Structure, Function, and Pathogenic Mechanisms

### *GBA1* gene regulation and transcript diversity

**Figure 2:**
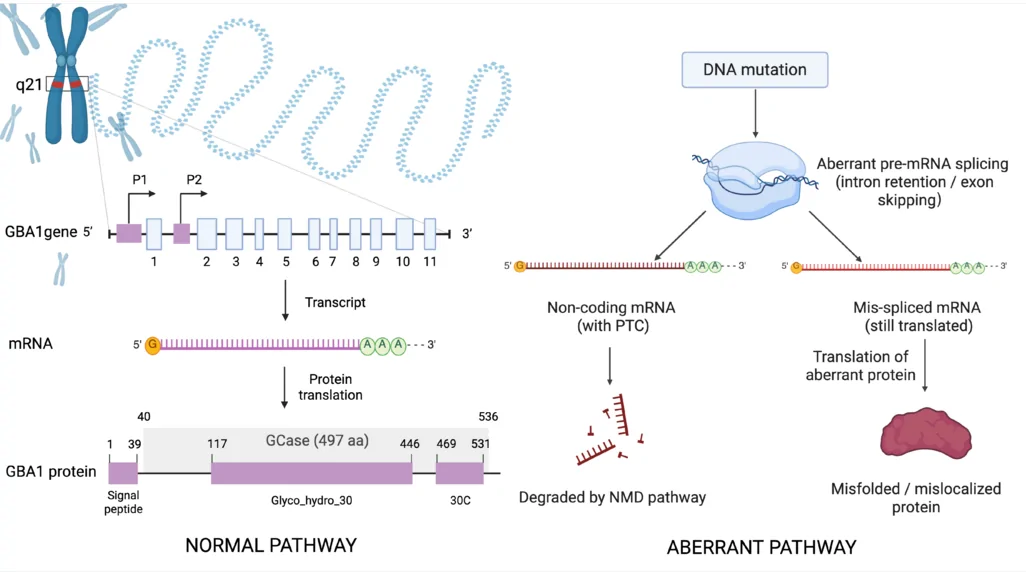
Canonical and aberrant splicing pathways of the *GBA1* gene. This diagram illustrates the transcriptional and translational outcomes of normal *versus* aberrant *GBA1* pre-mRNA splicing. On the left, canonical splicing produces a mature mRNA that is efficiently translated into functional GCase, which is properly folded, trafficked to lysosomes, and contributes to sphingolipid metabolism. On the right, mutations or dysregulation of splicing factors lead to abnormal splicing events, such as intron retention or exon skipping, resulting in two major outcomes: (1) generation of non-coding mRNA with PTCs, which are rapidly degraded by the NMD pathway; (2) production of misfolded or mislocalized GCase proteins from aberrant mRNAs, which are prone to ER retention and degradation, ultimately leading to reduced enzymatic activity. Created in BioRender. Wang, R. (2025) https://BioRender.com/6fyn84w. ER: Endoplasmic reticulum; GBA1: glucocerebrosidase gene; GCase: β-glucocerebrosidase; mRNA: messenger RNA; NMD: nonsense-mediated decay; PTC: premature termination codon.

The *GBA1* gene, located on chromosome 1q21, spans approximately 10 kilobases and comprises 11 exons (Behl et al., 2021; Zhang et al., 2024). It encodes GCase, a lysosomal hydrolase essential for sphingolipid metabolism (Yoon et al., 2024; Zhang et al., 2024). Alternative splicing of *GBA1* pre-mRNA generates different transcript variants (Behl et al., 2021; Granek et al., 2023), some of which lack coding capacity or harbor premature stop codons, rendering them targets for nonsense-mediated decay. Others encode protein isoforms with altered enzymatic efficiency or subcellular localization (Ging et al., 2024). Although their precise biological role remains under investigation, accumulating evidence suggests that transcript diversity may modulate GCase function and contribute to the phenotypic heterogeneity observed in *GBA1*-related disorders (Gustavsson et al., 2024; **Figure 2**).

Aberrant *GBA1* splicing patterns, either mutation-driven or due to dysregulated splicing factors, have been implicated in both Gaucher disease (GD) and PD (Hertz et al., 2024). For instance, intron retention and exon skipping reduce functional GCase levels, promoting GlcCer accumulation, a hallmark shared by GD and *GBA1*-PD (Zhang et al., 2024). Strategies to modulate splicing or enhance promoter activity are currently being explored to restore enzyme function (den Heijer et al., 2023a; Fregno et al., 2025; Okai et al., 2025). Furthermore, specific *GBA1* isoforms show promise as biomarkers for disease progression or treatment response (den Heijer et al., 2023b; Castillo-Ribelles et al., 2024). The gene’s complex transcriptional architecture, featuring dual promoters, alternative splicing, and a highly homologous pseudogene *GBAP1*, underscores its pivotal role in lysosomal biology and disease susceptibility.

Strategies to modulate splicing or enhance promoter activity are currently being explored to restore enzyme function (den Heijer et al., 2023a; Fregno et al., 2025; Okai et al., 2025). Furthermore, specific *GBA1* isoforms show promise as biomarkers for disease progression or treatment response (den Heijer et al., 2023b; Castillo-Ribelles et al., 2024). The gene’s complex transcriptional architecture, including dual promoters, alternative splicing, and the highly homologous pseudogene *GBAP1*, underscores its pivotal role in lysosomal biology and disease susceptibility.

### Glucocerebrosidase structure and function

GCase, encoded by *GBA1*, is a lysosomal glycoprotein (composed of 497 amino acids and structurally defined by three domains: a signal peptide for endoplasmic reticulum (ER) translocation, a central catalytic Glyco_hydro_30 domain (residues 40–536), and a C-terminal β-sandwich (Glyco_hydro_30C) domain critical for structural stability. The active site, sharing architectural features with acid ceramidases, facilitates the hydrolysis of GlcCer into glucose and ceramide, a key step in sphingolipid degradation and membrane homeostasis (Boer et al., 2020; Dal Maso et al., 2025).

Proper trafficking of GCase to the lysosome depends on its interaction with the lysosomal membrane protein *LIMP-2*. Mutations that disrupt this interaction, particularly within helix 5 and 7 of *LIMP-2*, lead to ER retention and degradation of misfolded enzyme (Dobert et al., 2025; Fregno et al., 2025). Structural modeling has revealed that variants affecting the GCase–SapC interface, such as E326K, impair cofactor binding and compromise enzymatic stability, even in the absence of catalytic site alterations (Lanore et al., 2025).

Clinically relevant variants such as *L444P* and *N370S* reduce enzymatic activity by 50%–70%, promoting accumulation of GlcCer and its deacylated counterpart, GlcSph, both implicated in GD and PD (Clemente et al., 2020; Perez-Abshana et al., 2024; Ciccaldo et al., 2025; Fregno et al., 2025). Beyond its canonical role in lipid catabolism, GCase participates in multiple cellular pathways, including autophagy, ER stress responses, and extracellular vesicle biology. Its bidirectional interaction with α-syn, where reduced GCase activity fosters α-syn aggregation, and aggregated α-syn impairs GCase trafficking, defines a pathological feedback loop central to PD pathogenesis (Cui et al., 2022; Wessler and Meisner-Kober, 2025). Therapeutic strategies aim to stabilize mutant GCase, such as with ambroxol, enhance wild-type activity, such as with *LTI-291*, or facilitate lysosomal targeting via *LIMP-2* modulation (Clemente et al., 2020; den Heijer et al., 2023a; Dobert et al., 2025; Fregno et al., 2025). These approaches underscore the therapeutic potential of the enzyme as a molecular hub linking lysosomal dysfunction and neurodegeneration.

### Pathogenic mechanisms of *GBA1* mutations and cellular pathology

More than 400 *GBA1* mutations have been reported, most of which are missense variants affecting the catalytic or structural integrity of GCase (Yoon et al., 2024; Zhang et al., 2024). Pathogenic mutations such as *N370S*, *L444P*, and *E326K* impair proper GCase folding and trafficking, resulting in decreased lysosomal enzymatic activity (Granek et al., 2023; Pietrafesa et al., 2024; Avenali et al., 2025). In both peripheral tissues and brain samples from *GBA1*-mutation carriers, GCase activity is significantly decreased, correlating with disease severity in GD and PD (Avenali et al., 2025).

Misfolded GCase accumulates in the ER, triggering the unfolded protein response and downstream pro-apoptotic signaling. This cellular stress contributes to neuronal vulnerability, particularly in dopaminergic populations. Notably, GCase deficiency initiates α-syn accumulation, while aggregated α-syn further impairs GCase function, establishing a deleterious cycle that amplifies proteostatic imbalance (Granek et al., 2023; Frattini et al., 2025). A study using *GBA1*-PD patient-derived midbrain organoids has demonstrated the formation of Lewy body-like inclusions (Frattini et al., 2025), while *in vivo* models combining *GBA1* mutations with α-syn overexpression exhibit exacerbated pathology and motor deficits (Mahoney-Crane et al., 2023; Lado et al., 2025). Moreover, reduced GCase activity facilitates α-syn fibril “seeding,” propagating misfolded aggregates across neuronal circuits (Henderson et al., 2020). Additionally, altered lipid metabolism, characterized by elevated GlcCer and GlcSph, contributes to membrane instability, impaired vesicular trafficking, and neuroinflammation (Bo et al., 2022; Lelieveld et al., 2022).

Importantly, *GBA1* mutations affect not only α-syn-dependent mechanisms. In *GBA1*^L444P/+^ mice, synaptic deficits in the hippocampus occur independently of α-syn, indicating broader neurophysiological consequences (Lado et al., 2025). Cognitive decline is frequently observed in *GBA1* carriers, often preceding motor symptoms, and may be linked to early hippocampal dysfunction (Mahoney-Crane et al., 2023; Roeben et al., 2024; Lado et al., 2025). At the cellular level, impaired lysosomal-mitochondrial crosstalk, oxidative phosphorylation deficits, and mitochondrial membrane depolarization have been documented, indicating a multifaceted disruption of neuronal homeostasis (Blumenreich et al., 2024; Perez-Abshana et al., 2024).

Recent large-scale genetic studies have refined our understanding of genotype-phenotype correlations in *GBA1*-PD (Ren et al., 2022; Koç Yekedüz et al., 2025; Rossi et al., 2025). Analyses of missense variants identified two principal components (PCs): PC1, associated with reduced enzyme activity and cognitive/motor decline, and PC2, linked to impaired Saposin C interaction and earlier disease onset (Lanore et al., 2025). These findings indicate that PD pathogenesis and clinical heterogeneity arise not only from loss of enzymatic function but also from disrupted protein-protein interactions, emphasizing the need for variant-specific therapeutic strategies.

**Figure 3:**
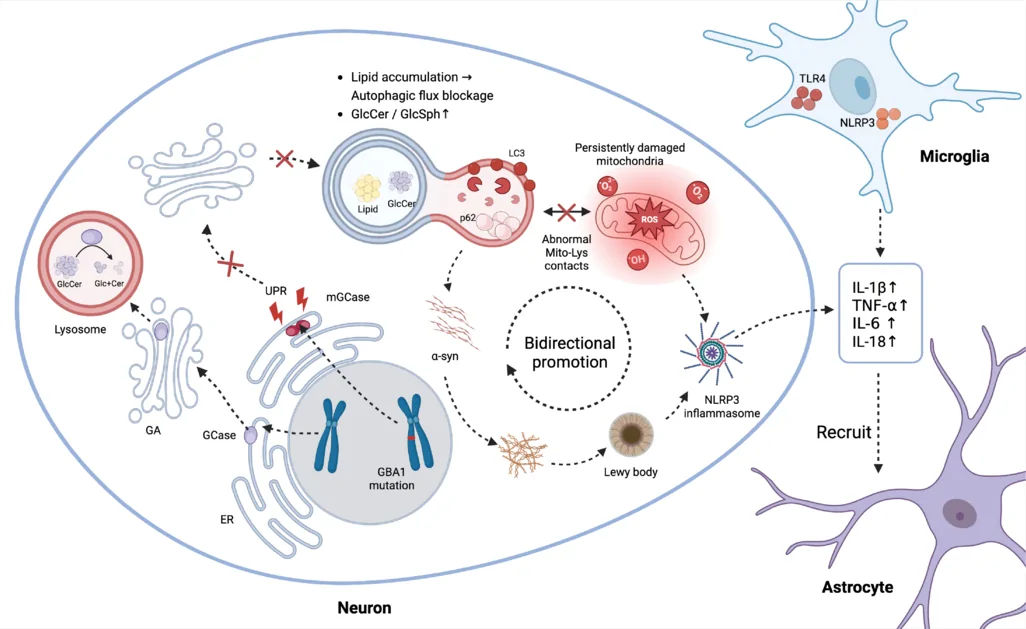
*GBA1* dysfunction impairs lysosomal function and autophagy, contributing to α-Syn accumulation and neurodegeneration. Mutations in *GBA1* lead to misfolded GCase proteins retained in the ER, triggering ER stress and the UPR. Reduced GCase activity decreases lysosomal degradation capacity, resulting in the accumulation of GlcCer and GlcSph, which in turn promote α-Syn aggregation. Aggregated α-Syn further impairs lysosomal function, blocks autophagic flux, and triggers neuroinflammation via microglial and inflammasome activation. These events collectively exacerbate neuronal damage and contribute to Parkinson’s disease pathogenesis. Created in BioRender. Wang, R. (2025) https://BioRender.com/gcku7t9. α-Syn: α-Synuclein; ER: endoplasmic reticulum; GBA1: glucocerebrosidase gene; GCase: glucocerebrosidase; GlcCer: glucosylceramide; GlcSph: glucosylsphingosine; IL: interleukin; PD: Parkinson’s disease; TNF: tumor necrosis factor; UPR: unfolded protein response.

Taken together, *GBA1* mutations drive a complex pathogenic cascade involving protein misfolding, lysosomal and mitochondrial dysfunction, and chronic inflammation, hallmarks shared across GD and PD. Elucidating these mechanisms is crucial for developing targeted therapies to restore GCase activity and halt neurodegenerative progression (**Figure 3**).

## The *GBA1* Gene: Cross-Disease Perspectives from Gaucher Disease to Parkinson’s Disease

### *GBA1* mutations in Gaucher disease and Parkinson’s disease

GD, the most common lysosomal storage disorder, is caused by biallelic mutations in *GBA1*, leading to GCase deficiency and pathological accumulation of GlcCer and GlcSph, particularly in macrophage-lineage cells (Basgalupp et al., 2023; Liu et al., 2023; Marano et al., 2024). GD manifests with considerable phenotypic heterogeneity: type 1 is non-neuronopathic and primarily visceral, whereas types 2 and 3 involve progressive central nervous system (CNS) degeneration, with GlcSph levels correlating with neurological severity (Marano et al., 2024; Grabowski et al., 2025).

Heterozygous *GBA1* mutations, especially *L444P*, *N370S*, and *E326K*, are now recognized as the most significant genetic risk factor for PD, increasing disease susceptibility up to 10-fold (Marano et al., 2024; Zhang et al., 2024). Risk stratification mirrors mutation severity: severe variants such as *L444P* and *RecNciI* are associated with earlier onset, faster progression, and higher dementia risk, while mild or risk variants such as *E326K* and *T75del* confer intermediate phenotypes (den Heijer et al., 2020; Parlar et al., 2023; Skrahin et al., 2024). In a large Turkish cohort, the prevalence of *GBA1* variants in PD patients was 13.2%, significantly higher than in controls (6.4%), with p.T369M being the most frequent variant (19.1%), a variant previously considered benign (Koç Yekedüz et al., 2025).

Notably, male *GBA1*-PD patients show accelerated cognitive decline compared to females, implicating sex-specific modifiers (Caminiti et al., 2025). This sexual dimorphism extends beyond clinical presentation to underlying mechanisms, as microglial responses to GCase deficiency exhibit significant sex differences. Female microglia demonstrate greater sensitivity to glucocerebrosidase inhibition, with more pronounced impairment of neuroprotective functions, including nuclear factor E2-related factor 2 (Nrf2) pathway activation (Brunialti et al., 2021). These findings may explain the attenuated female protection typically observed in idiopathic PD, as the 1.5–2-fold reduced risk in females is lost in GBA1-PD populations (Li et al., 2021a).

Mechanistically, GCase deficiency impairs lysosomal degradation of α-syn, promoting Lewy body formation and toxic aggregate propagation (Granek et al., 2023; Skrahin et al., 2024). This is compounded by disruption of the autophagy-lysosome axis, driven by dysregulation of transcription factor EB (*TFEB*) and mammalian target of rapamycin (mTOR) pathways (Lopez et al., 2022; Marano et al., 2024). Accumulation of GlcSph not only destabilizes membrane homeostasis but also activates microglia, triggering chronic neuroinflammation (Marano et al., 2024; Skrahin et al., 2024; Slingerland et al., 2025). These converging mechanisms underlie both GD neuropathology and *GBA1*-PD.

The shared molecular landscape across GD and PD, defined by lipid accumulation, lysosomal impairment, and proteostatic stress, positions GCase at the intersection of metabolism and neurodegeneration. As such, enhancing GCase function or modulating lipid metabolism represents a rational therapeutic avenue for disease modification.

### Clinical features and ethnic variability in *GBA1*-Parkinson’s disease

The clinical presentation of *GBA1*-PD is influenced by both the type and severity of the GBA1 mutation, as well as ethnic background. Remarkably, different populations exhibit distinct mutation spectra: *L444P*/*L483P* mutation predominates in East Asian populations (China/Japan, 5%–10% prevalence), while *N370S* is exceptionally rare (Zhou et al., 2023). Ashkenazi Jewish cohorts show high frequencies of *N370S* (15%–20%) and *E326K* (Jia et al., 2022; Koros et al., 2024). Unique variants have also been identified in other groups, such as the p.T369M mutation in Turkish patients (Koç Yekedüz et al., 2025), and the *rs3115534* variant in African populations, while Latin American groups exhibit region-specific mutations such as *K198E* in Colombians (Koros et al., 2024).

The current classification of *GBA1* mutations is based on the three types of GD. The neuronopathic type-causing *GBA1* mutations, such as *L444P*, *84GG*, and *D409H*, are collectively called “severe” mutations. The T1GD-causing mutations, such as *N370S*, are known as “mild” mutations. *GBA1* mutations that increase the risk of PD in carriers but do not cause GD when present bi-allelically, such as *E326K* and *T369M*, are called “risk” mutations or variants (Yoon et al., 2024). Severe mutations reduce activity to 13%–24%, while mild, such as *N370S*, or benign variants, such as *E326K*, retain over 50% activity (Navarro-Romero et al., 2022; Smith and Schapira, 2022). These differences translate into distinct clinical trajectories. For example, with severe variants (*L444P*/*L483P*) causing earlier onset (mean age 50 years *versus* 54 years in sporadic PD) and accelerated cognitive decline (HR=3.15) (Zhou et al., 2023; Orrú et al., 2025). Carriers of *E326K*, previously considered benign, show comparable cognitive impairment and more rapid motor decline than idiopathic PD patients (Menozzi et al., 2023).

Clinically, *GBA1*-PD presents as an aggressive PD subtype. Onset is typically 3.5–5 years earlier than idiopathic PD, with 70% of cases developing before age 50. Patients require higher levodopa equivalent daily doses and experience more frequent motor complications and earlier development of dementia (Parlar et al., 2023; Skrahin et al., 2024). Non-motor symptoms, including depression, psychosis, rapid eye movement sleep behavior disorder, and autonomic dysfunction, are also more pronounced. Mortality risk is significantly increased (1.8–2.5×), underscoring the need for tailored clinical management (Bentley-DeSousa et al., 2025).

Notably, the clinical impact of mild and risk variants is increasingly recognized, suggesting variant-specific pathogenic mechanisms that necessitate a precision medicine approach. Kinetic biomarkers such as TTT enable early intervention by identifying high-risk subgroups (Orrú et al., 2025), while pharmacogenomic insights reveal suboptimal DBS responses in *GBA1*-PD (Bressan et al., 2023). Genotyping thus optimizes therapeutic selection, with variant-specific approaches improving outcomes in this PD subgroup (**Table 1**). Their underlying molecular mechanisms are explored in depth in Section Therapeutic Strategies Targeting *GBA1* and Neuroinflammation.

**Table 1:** Clinical characteristics and therapeutic strategies for common *GBA1* variants

Mutation type	Residual GCase activity	Age of onset (yr)	Major clinical phenotype	Recommended treatment strategy	Notes (Advantages/limitations)	Clinical trial phase
*L444P* (severe)	13%–24%	40–50	Rapidly progressing motor and cognitive impairments	Ambroxol + anti-IL-1β therapy + gene therapy (AAV9)	Rapid onset of action but fast disease progression	Phase II/III (Hwang et al., 2024)
*N370S* (mild)	30%–50%	50–60	Moderate motor symptoms, relatively preserved cognition	Ambroxol monotherapy ± probiotic intervention	Good efficacy, slow progression	Phase II (Elstein et al., 2017)
*E326K* (benign)	> 50%	After 60	Mainly mild motor symptoms	Probiotic treatment + lifestyle intervention	Requires long-term monitoring, slow progression	Observational stage

The residual GCase activity, age of onset, clinical phenotypes, recommended treatment strategies, and clinical trial stages are associated with L444P, N370S, and E326K mutations.

### Shared autophagy and lysosomal pathology in genetic forms of Parkinson’s disease

Despite their distinct genetic origins, PD-related mutations in *GBA1*, *LRRK2*, and *PINK1*/*Parkin* converge on a common pathogenic axis: disruption of autophagy–lysosomal pathway (ALP) and impaired proteostasis, ultimately leading to α-syn accumulation and neuronal dysfunction (Pang et al., 2022; Volta, 2023; Narendra and Youle, 2024; Bentley-DeSousa et al., 2025). Recent data further show that *GBA1* variants with high PC2 scores, which are indicative of disrupted SapC binding, are associated with impaired chaperone-mediated autophagy and α-syn accumulation, independent of catalytic loss (Lanore et al., 2025).

In *GBA1*-PD, *loss-of-function* mutations reduce GCase activity, impair lysosomal degradation of α-syn, and promote GlcSph-driven neuroinflammation (Pang et al., 2022; Volta, 2023). Conversely, *LRRK2* mutations exert *gain-of-function* effects through hyperactive kinase signaling, altering Rab GTPase-dependent lysosomal trafficking, autophagosome maturation, and chaperone-mediated autophagy (Albanese et al., 2021; Volta, 2023). In *PINK1*/*Parkin*-related PD, defective mitophagy arises from impaired sensing and clearance of damaged mitochondria, disrupting mitochondrial quality control and potentially impacting lysosomal function upstream of lysosomal degradation (Ge et al., 2020; Narendra and Youle, 2024).

These diverse mutations yield a shared phenotype of α-syn accumulation, lysosomal stress, such as impaired acidification, reduced hydrolase activity, altered morphology, and secondary inflammation (Pang et al., 2022; Volta, 2023). Restoration of lysosomal homeostasis has thus emerged as a unifying therapeutic strategy. Approaches such as TFEB activation to enhance lysosomal biogenesis, GCase chaperoning, such as with ambroxol, or LRRK2 kinase inhibition show efficacy in multiple PD models, including those harboring *GBA1* or *LRRK2* mutations (Xue et al., 2023; Yang et al., 2023).

These findings underscore a broader principle in PD pathogenesis: genetic heterogeneity funnels into convergent cellular dysfunction. Targeting shared downstream pathways, rather than gene-specific defects, may thus offer the most effective strategy for disease modification across genetic PD subtypes.

## *GBA1* Dysfunction and Neuroinflammation

### α-Synuclein aggregation and inflammasome activation

*Loss-of-function* mutations in *GBA1*, which encodes GCase, are among the strongest genetic risk factors for PD, linking lysosomal dysfunction to protein aggregation and neuroinflammation. In physiological conditions, GCase contributes to the breakdown of GlcCer and other sphingolipids within lysosomes. Deficient GCase activity, as observed in *GBA1*-PD, disrupts lysosomal homeostasis, resulting in the accumulation of undegraded lipids and impaired chaperone-mediated autophagy. This leads to reduced clearance of misfolded proteins, particularly α-syn (Granek et al., 2023; Dai et al., 2024).

GCase deficiency promotes the formation of toxic α-syn oligomers and fibrils by impairing lysosomal degradation and increasing lipid substrate burden. In turn, pathological α-syn aggregates bind directly to GCase at the lysosomal surface and inhibit its enzymatic activity, perpetuating a feedforward cycle of proteinopathy and lysosomal stress (Granek et al., 2023; Dai et al., 2024). This reciprocal interaction is a key molecular axis through which *GBA1* mutations accelerate the development of Lewy pathology.

In addition to driving protein aggregation, α-syn fibrils elicit potent immune responses in the brain. These misfolded assemblies activate microglia via pattern recognition receptors, including Toll-like receptor 2 (TLR2) and TLR4, triggering canonical nuclear factor kappa B (NF-κB) signaling and transcription of pro-inflammatory mediators such as tumor necrosis factor alpha (TNF-α), interleukin (IL)-1β, and IL-6 (Soraci et al., 2023). Aggregated α-syn also serves as a danger-associated molecular pattern for the *NLRP3* inflammasome, a cytosolic platform that governs caspase-1 activation and the maturation of IL-1β and IL-18 (Li et al., 2021b; Huang et al., 2023). Mitochondrial reactive oxygen species (ROS) generated during this process further damages organelles and sensitizes dopaminergic neurons to degeneration (Huang et al., 2023; Abdelaziz, 2025). Recent reviews emphasize NLRP3 as a convergent therapeutic target across *GBA1* mutation subtypes. Newer inhibitors, such as RRx-001 and NT-0796, demonstrate improved blood–brain barrier (BBB) penetration and reduced hepatotoxicity compared with MCC950 (Haque et al., 2024).

Animal models provide compelling evidence that *GBA1* loss amplifies this inflammatory cascade. *GBA1* heterozygous or knockout mice show early and sustained accumulation of α-syn, along with pronounced microgliosis and dopaminergic loss in the substantia nigra *pars compacta* (Kuo et al., 2022). In toxin-based models, such as MPTP exposure, mice harboring *GBA1* mutations (e.g., *GBA*^+/L444P^) exhibit greater vulnerability, with elevated cytokine production, exacerbated oxidative stress, and enhanced nigrostriatal damage compared to wild-type controls (Yun et al., 2018). Together, these findings indicate that GCase deficiency creates a permissive environment for α-syn pathology and neuroinflammation, acting as a molecular bridge between lysosomal dysfunction and innate immune activation.

### Astrocyte-mediated neurotoxicity

Astrocytes, the most abundant glial population in the CNS, play critical roles in synaptic regulation, metabolic support, and immune modulation. In the context of *GBA1*-PD, growing evidence indicates that astrocyte dysfunction contributes actively to neurodegeneration through both *loss-of-function* and toxic *gain-of-function* mechanisms (Wang et al., 2021).

Under normal conditions, astrocytes maintain glutamate homeostasis by clearing excess neurotransmitter via *EAAT2* (GLT-1) transporters, support neuronal metabolism through lipid storage and energy transfer, and secrete neurotrophic factors such as glial cell-derived neurotrophic factor (Wang et al., 2021). However, GCase deficiency disrupts these essential functions. Studies using primary mouse astrocytes and iPSC-derived human models have shown that *GBA1* mutations impair lysosomal clearance and lipid metabolism, leading to the intracellular accumulation of GlcCer, sphingomyelin, and other lipids, as well as abnormal lipid droplet morphology (Sanyal et al., 2020; Gregorio et al., 2024; Tahanis et al., 2025). This metabolic imbalance not only limits astrocytic support to neurons but also contributes to α-syn propagation by interfering with vesicular trafficking and degradation (Bo et al., 2022).

Concomitant reductions in GLT-1 expression result in excessive extracellular glutamate, increasing the risk of excitotoxicity and synaptic dysfunction (Sanyal et al., 2020). Importantly, single-cell RNA sequencing analyses in both mouse models and human iPSC-derived astrocytes reveal complex transcriptional alterations, extending beyond the canonical A1/A2 dichotomy. These include dysregulation of lysosomal genes, innate immune pathways (e.g., NF-κB signaling), and antigen presentation modules (D’Sa et al., 2025). While many *GBA1*-mutant astrocytes exhibit a hypoinflammatory phenotype, characterized by blunted TLR4 signaling and reduced cytokine release (Ding et al., 2021), other *GBA1* variants (e.g., *D409V* and *E326K*) have been associated with paradoxical pro-inflammatory responses, including CCL2 overproduction and amplification of microglial activation (Bo et al., 2022; Kweon et al., 2024b).

Beyond inflammatory crosstalk, astrocytes also contribute to non-cell-autonomous neuronal toxicity via release of exosomes containing misfolded α-syn (Rademacher, 2023), impairment of the antioxidant Nrf2 pathway (Brunialti et al., 2021), and failure to maintain metabolic resilience. These mechanisms have been shown to compromise the survival of dopaminergic neurons in midbrain organoids and *in vivo* models (Kim et al., 2021b; Nam et al., 2025).

Although most of these findings derive from preclinical systems, including murine models, fly models, and iPSC-derived glial cultures, they collectively highlight astrocytes as central players in the pathophysiology of *GBA1*-PD. Their role transcends passive response and encompasses active promotion of neurodegeneration through multifaceted mechanisms.

### Gut–brain axis and microbiome dysbiosis

Emerging evidence implicates the gut–brain axis as a key contributor to the pathogenesis of *GBA1*-PD (Cossu et al., 2023). Alterations in the composition and function of the intestinal microbiota have been consistently reported in both patients and animal models carrying *GBA1* mutations, suggesting a link between lysosomal dysfunction, intestinal inflammation, and central neurodegeneration (Atilano et al., 2023; Kearns, 2024).

In Gba1b-deficient Drosophila models, 16S rRNA sequencing analysis of gut microbiota revealed significant dysbiosis, characterized by increased abundance of Acetobacter and Lactobacillus genera compared to wild-type controls. This shift was associated with elevated total bacterial load and chronic immune activation (Atilano et al., 2023). These changes correlate with impaired intestinal barrier function, evidenced by increased gut permeability and dysregulation of tight junction integrity, leading to elevated circulating levels of microbial products, such as lipopolysaccharide (LPS) (Kearns, 2024). Translocation of gut-derived microbial metabolites into the bloodstream triggers systemic immune activation. These signals propagate to the CNS through neural, endocrine, and inflammatory pathways, where metabolites such as LPS, bile acids, and tryptophan catabolites directly modulate microglial function via receptors including TLR4, GPBAR1/TGR5, and AhR, amplifying neuroinflammation (Deng et al., 2024).

GBA1-deficient animals exhibit significantly elevated expression of pro-inflammatory cytokines (IL-6, TNF-α) in both gut and brain tissue, accompanied by progressive motor deficits (Atilano et al., 2023), consistent with microglia-mediated neuronal dysfunction through NF-κB/STAT3 signaling pathways (Kearns, 2024). Conversely, germ-free *GBA1*-KO mice display attenuated microglial activation and reduced α-syn aggregation, directly implicating gut-derived microbial metabolites (e.g., LPS and TMAO) as drivers of neuroinflammation via TLR4/ROS-dependent cascades (Atilano et al., 2023; Deng et al., 2024; Kearns, 2024).

Sex-specific differences in neuroinflammatory responses further modulate *GBA1*-PD pathogenesis. Microglial morphological and functional analyses reveal that GCase inhibition induces more dramatic phenotypic changes in female microglia, reducing their superior neuroprotective capacity to levels comparable to male microglia (Brunialti et al., 2021). This differential microglial response may contribute to the loss of female protection typically seen in idiopathic PD, resulting in a more balanced sex distribution in *GBA1*-PD cohorts (Li et al., 2021a).

SCFAs, such as gut microbiota-derived butyrate, modulate immunity via HDAC inhibition and GPCR activation, thereby promoting *Treg* differentiation (↑FoxP3^+^) to suppress inflammation (Deng et al., 2024; Kearns, 2024). Critically, *GBA1* mutations, which disrupt lysosomal GCase function, also impair microbial homeostasis, leading to a reduction of over 50% in fecal butyrate levels (Atilano et al., 2023). This deficit directly triggers microglial activation/neuroinflammation, worsening motor deficits in *GBA1*-linked neurodegeneration. Fecal microbiota transplantation counters *GBA1*-driven dysbiosis by replenishing SCFA-producers, ameliorating neuroinflammation (Yi et al., 2025).

Therapeutic interventions targeting the gut microbiota have shown efficacy in preclinical models. Supplementation with *Lactobacillus plantarum* PS128 enhances gut barrier integrity, reduces microglial activation, and improves motor function by modulating gut-brain axis signaling (Atilano et al., 2023). Similarly, washed microbiota transplantation (an optimized fecal microbiota transplantation protocol) restores microbial diversity (Shannon index↑), attenuates α-syn pathology (phosphorylated α-syn↓), and rescues behavioral deficits in PD models (Yi et al., 2025).

**Figure 4:**
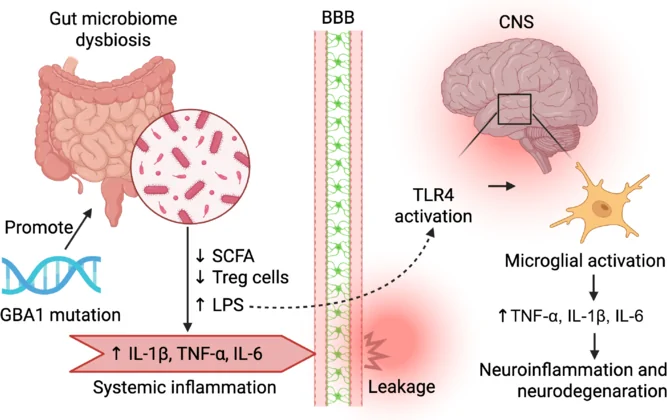
Schematic representation of the gut–brain axis involvement in *GBA1*-associated neurodegeneration. *GBA1* deficiency induces gut microbiota dysbiosis, characterized by reduced levels of beneficial bacteria and SCFAs, along with decreased Treg cell differentiation. This dysregulation leads to elevated levels of microbial-derived LPS and increased production of pro-inflammatory cytokines, resulting in impaired intestinal and BBB integrity. Translocated LPS activates microglial TLR4 signaling in the brain, triggering microglial activation, neuroinflammation, and progressive neurodegenerative changes. Created in BioRender. Wang, R. (2025) https://BioRender.com/uyqa0u7. BBB: Blood–brain barrier; CNS: central nervous system; GBA1: glucosylceramidase beta 1; IL: interleukin; LPS: lipopolysaccharide; SCFAs: short-chain fatty acids; TLR4: Toll-like receptor 4; TNF: tumor necrosis factor; Treg: regulatory T cell.

Despite these promising results, critical questions remain. It is still unclear whether microbiome alterations precede neurodegeneration or arise as a consequence of systemic metabolic stress in *GBA1*-PD. Moreover, robust clinical studies are needed to validate the therapeutic potential of microbiome manipulation in human *GBA1* carriers. Nonetheless, the gut–brain axis represents a tractable and potentially modifiable pathway in the search for disease-modifying interventions in PD (**Figure 4**).

### Oligodendrocytes and myelin homeostasis

Oligodendrocytes, the CNS cells responsible for axonal myelination and metabolic support, are pivotal for maintaining white matter integrity (Morrison et al., 2013). In the context of *GBA1* deficiency, accumulating evidence suggests that oligodendrocyte dysfunction is not merely a consequence of intrinsic lipid vulnerabilities but may actively contribute to neuroinflammation and neurodegeneration (Boddupalli et al., 2022; Gregorio et al., 2024).

Under physiological conditions, oligodendrocyte precursor cells (OPCs) differentiate into mature myelinating oligodendrocytes, sustaining myelin turnover and providing neurons with lactate and lipids (Asadollahi et al., 2024). Loss of GCase activity impairs lysosomal sphingolipids degradation, causing the accumulation of bioactive lipids such as GlcCer and psychosine, which disrupt OPC differentiation and compromise myelin stability (Gregorio et al., 2024). In Gba1^f/f::cre mice, GCase deficiency impairs maturation of myelinating oligodendrocytes and induces lipid metabolic imbalance in white matter, consistent with *in vitro* studies using CBE, a selective GCase inhibitor, which mimics enzymatic deficiency (Gregorio et al., 2024).

Proper lysosomal acidification is essential for OPCs differentiation. Lysosomes in heterozygous and homozygous *GBA1* mutant astrocytes are significantly alkalinized (pH ~6) compared with WT controls, suggesting that *GBA1* deficiency disrepts glial functions, including OPCs (Sanyal et al., 2020). In HIV-associated neurocognitive disorder models, the antiretroviral drug bictegravir inhibits OPC differentiation via lysosomal alkalinization, an effect reversible by TRPML1 activation (Festa et al., 2023).

Collectively, these findings indicate that lysosomal dysfunction, whether from enzymatic deficiency or pharmacological interference, plays a central role in impaired myelination and white matter dysfunction. Clinically, both neuropathic and non-neuropathic GD patients show white matter abnormalities, and similar damage in PD patients correlates with motor deficits, emphasizing the pivotal role of oligodendrocytes in neurodegeneration (Gregorio et al., 2024).

**Figure 5:**
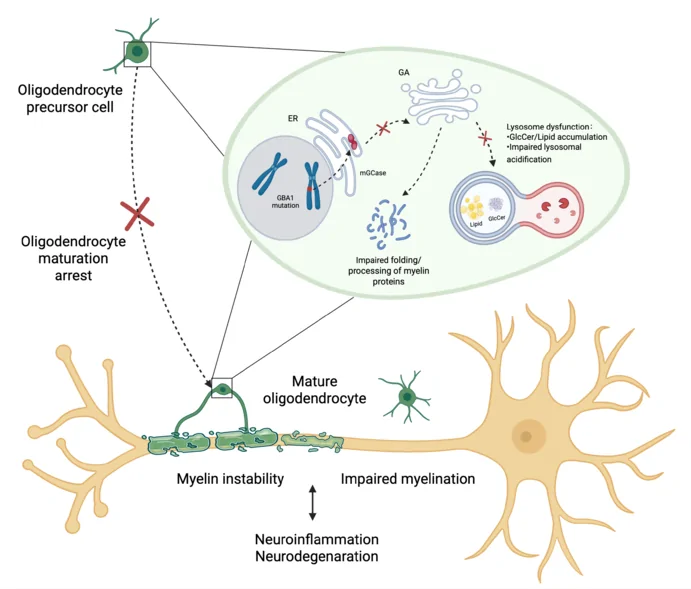
Mutations in *GBA1* lead to reduced activity of GCase, resulting in lysosomal dysfunction. The impaired degradative capacity of lysosomes causes abnormal accumulation of GlcCer and other sphingolipids within lysosomes. This lipid overload not only disrupts the differentiation of oligodendrocyte precursor cells into mature oligodendrocytes but also impairs the folding and processing of myelin-associated proteins, such as myelin basic protein and proteolipid protein, thereby leading to defective myelination and destabilization of myelin structures. These myelin abnormalities synergize with neuroinflammatory responses, ultimately accelerating neurodegenerative processes. Created in BioRender. Wang, R. (2025) https://BioRender.com/2gu6hct. ER: Endoplasmic reticulum; GA: Golgi apparatus; GBA1: glucosylceramidase beta 1; GCase: glucocerebrosidase; GlcCer: glucosylceramide; mGCase: mutant glucocerebrosidase.

In summary, oligodendrocytes are active contributors in *GBA1*-related pathology, driving neurodegeneration through lipid dysregulation, inflammatory amplification, and propagation of α-syn pathology (**Figure 5**).

## Molecular Crosstalk between *GBA1* and Neurodegeneration

### Mitochondrial dysfunction

*GBA1* mutations profoundly impair mitochondrial homeostasis, contributing critically to GBA1-linked neurodegeneration (Kim et al., 2021a; Rubilar et al., 2024). GCase deficiency disrupts mitochondrial dynamics, leading to excessive fragmentation, impaired adenosine triphosphate production, and increased oxidative stress (Smith and Schapira, 2022). These alterations are compounded by mitochondrial DNA damage and impaired mitophagy, particularly via *PINK1*/*Parkin* downregulation, which together promote α-syn aggregation and neuronal death (Li et al., 2023; Cossu and Hattori, 2024).

Beyond its lysosomal localization, GCase is also present within mitochondria, where it modulates complex I integrity and mitochondrial proteostasis through interactions with proteins such as *HSP60* and *LONP1* (Shin et al., 2021). *GBA1* mutations destabilize complex I, further limiting oxidative phosphorylation and augmenting ROS generation (Nam et al., 2024). Dysfunctional mitochondria accumulate as mitophagic clearance fails, reinforcing a self-perpetuating loop of cellular stress (Li et al., 2023; Rubilar et al., 2024).

Mitochondria–lysosome crosstalk is another critical node affected by GCase deficiency. *TBC1D15*-mediated Rab7 GTP hydrolysis is impaired, prolonging mitochondria-lysosome contacts and disrupting mitochondrial fission and lysosomal function (Kim et al., 2021a). Meanwhile, lysosomal lipid accumulation, particularly GlcCer, hampers autophagic clearance and drives further α-syn aggregation (Henderson et al., 2020; Smith and Schapira, 2022). Dopaminergic neurons are especially vulnerable, as dopamine metabolism generates quinones that inhibit GCase and exacerbate mitochondrial stress (Henderson et al., 2020).

Calcium dyshomeostasis further links *GBA1* to mitochondrial toxicity. Mutant GCase alters *VDAC1* function and ER-mitochondrial calcium exchange, weakening calcium buffering and increasing susceptibility to excitotoxicity (Argueti-Ostrovsky et al., 2024). *In vivo*, these defects manifest as elevated mitochondrial ROS and increased α-syn misfolding, reinforcing neurodegenerative cascades (Nam et al., 2024). Therapeutically, targeting mitophagy (e.g., via TFEB or mTORC1 modulation), oxidative stress (e.g., coenzyme Q10), or calcium signaling offers avenues to mitigate mitochondrial dysfunction in *GBA1*-PD. GCase chaperones such as ambroxol also show promise in restoring mitochondrial integrity via complex I stabilization and lysosomal- mitochondrial repair (Rubilar et al., 2024).

### Autophagy–lysosomal pathway impairment

The ALP is essential for proteostasis, enabling degradation of misfolded proteins and damaged organelles (Pradas and Martinez-Vicente, 2023). *GBA1* mutations compromise this system at multiple levels. Lysosomal dysfunction impairs autophagosome-lysosome fusion, resulting in the accumulation of autophagosomes (marked by LC3-II) and the autophagy adaptor p62/SQSTM1, indicative of stalled autophagic flux (Gallagher and Holzbaur, 2023). These defects facilitate toxic α-syn accumulation and propagation, while chaperone-mediated autophagy failure further impairs α-syn clearance (Navarro-Romero et al., 2022; Pradas and Martinez-Vicente, 2023).

The ALP is also disrupted by GlcCer accumulation, which alters lysosomal membrane composition and destabilizes α-syn homeostasis (Bo et al., 2022; Lunghi et al., 2022). Misfolded GCase entrapped within lysosomes triggers proteostatic stress, compounding autophagic clearance failure and disrupting mitochondrial-lysosomal communication. This impairment in mitophagic flux undermines organelle quality control and enhances neuronal susceptibility to proteotoxic stress (Smith and Schapira, 2022).

Interestingly, ALP dysfunction varies by *GBA1* mutation. The common *N370S* variant is associated with mTOR hyperactivation and lysosomal degradation defects, while *L444P* carriers show paradoxical increases in autophagic flux. These divergent responses highlight the need for variant-specific therapeutic strategies (Seo et al., 2021; Sahyadri et al., 2022; Bezrukova et al., 2025).

Although *TFEB*, a master regulator of lysosomal biogenesis, is upregulated in response to GCase deficiency, this compensatory mechanism is often insufficient. Persistent lipid overload and oxidative stress blunt the efficacy of *TFEB*-driven lysosomal restoration (Yang et al., 2023). Thus, ALP failure in *GBA1*-PD reflects the convergence of lipid dyshomeostasis, neuroinflammation, and mitochondrial dysfunction, underscoring the necessity of multi-targeted interventions (Jiao et al., 2025a).

### Epigenetic modifications

Epigenetic regulation critically influences *GBA1* expression and function. Hypermethylation of CpG islands within the *GBA1* promoter reduces transcriptional activity and correlates with decreased GCase levels in PD brains. In contrast, increased histone acetylation promotes *GBA1* expression, and histone deacetylase inhibitors, such as vorinostat or sodium butyrate, have shown promise in enhancing GCase activity through chromatin remodeling (Toker et al., 2021; Huh et al., 2023; Ging et al., 2024).

Non-coding RNAs further fine-tune *GBA1* expression. For instance, *miR-155* and *miR-214* modulate autophagy-related genes and are dysregulated in *GBA1*-PD, contributing to impaired proteostasis (Zhang et al., 2022; Hussain et al., 2024). Moreover, crosstalk between *GBA1* dysfunction and *SNCA* regulation reinforces pathogenic loops: DNA hypomethylation in *SNCA* intron 1 increases α-syn expression, while GCase deficiency promotes lysosomal failure and glycosphingolipid accumulation, which may themselves impact epigenetic landscapes (Brooker and Krainc, 2021; Smith et al., 2023).

Mitochondrial dysfunction resulting from *GBA1* mutations intensifies oxidative stress, which in turn alters DNA methylation patterns and perpetuates α-syn overexpression. This bidirectional relationship between enzymatic deficits and epigenetic regulation presents both a mechanistic insight and a therapeutic opportunity. Targeting epigenetic modifiers, such as histone deacetylase inhibitors, or restoring lysosomal function through GCase chaperones may offer synergistic benefits. Precision medicine approaches that integrate genetic, epigenetic, and metabolic profiles are likely to optimize therapeutic outcomes in *GBA1*-associated synucleinopathies (Zhang et al., 2024).

## Therapeutic Strategies Targeting *GBA1* and Neuroinflammation

### Enzyme replacement therapy and substrate reduction therapy

Although enzyme replacement therapy (ERT) has improved systemic manifestations in GD, its efficacy in *GBA1*-PD is limited by poor BBB penetration (Huh et al., 2023; Grabowski et al., 2025). To overcome this, strategies such as molecularly engineered GCase variants (e.g., GCase85), brain-targeted peptide conjugates (e.g., RVG and TfR), and nanoparticle-based formulations are under development. Intrathecal and intranasal delivery approaches are also being explored to bypass the BBB, with promising preclinical results supporting improved CNS targeting and α-syn clearance (Behl et al., 2021; Liang et al., 2024; Grabowski et al., 2025). TfR-fusions and nanocarrier ERTs are now in investigational new drug-enabling studies (Grabowski et al., 2025).

In parallel, brain-penetrant small molecule therapies that reduce substrate biosynthesis provide a complementary oral approach. The glucosylceramide synthase inhibitor venglustat, combined with imiglucerase in adults with GD type 3, was well tolerated, markedly lowered GlcCer and GlcSph levels in cerebrospinal fluid, and showed preliminary evidence of clinical stability, along with encouraging signals of improved brain connectivity on functional MRI (Schiffmann et al., 2023).

### Small molecule chaperones

Chaperone molecules aim to stabilize mutant GCase, restoring its trafficking and activity. Ambroxol, the most clinically advanced candidate, improves lysosomal GCase function, reduces α-syn burden, and has shown favorable outcomes in early-phase clinical trial settings (Mullin et al., 2020; Colucci et al., 2023). A recent registry study (*n* = 150) reports that high-dose ambroxol (median 435 mg/day) stabilizes motor symptoms in *GBA1*-PD patients over 19 months (Istaiti et al., 2025). However, efficacy varies by variant: p.N370S carriers showed 40% enzyme activity restoration *versus* 15% for p.L444P (Lanore et al., 2025), highlighting the need of genotype-guided dosing. Novel chaperones, such as NCGC326, display improved BBB permeability and selectivity (Williams et al., 2024).

Allosteric activators provide a complementary approach to directly enhance GCase catalytic function. LTI-291, a CNS-penetrant activator, was evaluated in a first-in-human study (up to 90 mg single, 60 mg multi-day) and a Phase 1B trial in *GBA1*-PD patients (10–60 mg daily for 28 days). It demonstrated good tolerability, dose-proportional pharmacokinetics, and adequate central exposure. A transient increase in intracellular glucosylceramide suggested target engagement via enhanced substrate flux, indicating modulation of glycosphingolipid metabolism. Long-term clinical relevance remains under investigation (den Heijer et al., 2021, 2023a).

Additional small molecules that indirectly modulate GCase activity or related pathways are under investigation. For instance, LRRK2 kinase inhibitors may influence lysosomal function, though their effect on GCase appears context dependent. One study found that the LRRK2 inhibitor MLi-2 did not increase GCase activity in macrophages from GD patients (Usenko et al., 2025).

Overall, challenges remain for all these strategies, including achieving mutation-specific efficacy, optimizing CNS delivery, and demonstrating long-term clinical benefits in larger trials (**Table 2**).

**Table 2:** Selected clinical trials of emerging therapeutic strategies for *GBA1*-PD

Therapeutic strategy	Compound(s)	Mechanism of action	Clinical trial phase	Key findings	Challenges/limitations
Pharmacological chaperones	Ambroxol	Stabilizes mutant GCase, enhances lysosomal trafficking	Phase II	High-dose (median 435 mg/d) stabilized motor symptoms over 19 mon in GBA1-PD patients (Istaiti et al., 2025); Variant-specific efficacy (N370S > L444P) (Lanore et al., 2025)	Mutation-specific efficacy, limited BBB penetration for some analogs
	NCGC326	Allosteric modulator	Preclinical	Enhanced BBB permeability and selectivity (Williams et al., 2024)	Early development stage
Allosteric activators	LTI-291 (BIA-28-6156)	Allosteric activation of wild-type and mutant GCase	Phase 1B	Well tolerated in healthy volunteers (SAD/MAD) and GBA1-PD patients (28-day); Dose-proportional PK; Good CNS penetration; Transient increase in PBMC GluCer levels (den Heijer et al., 2021, 2023b)	Transient GluCer increase mechanism unclear; Long-term efficacy unknown
NLRP3 inhibitors	MCC950	Selective NLRP3 inflammasome inhibition	Phase II	Reduced IL-1β and motor deficits in murine models; Hepatotoxicity risks and poor CNS penetration in humans (Wu et al., 2016; Li et al., 2021a, 2022)	Limited BBB penetration, hepatotoxicity
	RRx-001, NT-0796	NLRP3 inhibition with improved BBB penetration	Preclinical/Phase I	Improved BBB permeability compared to MCC950 (Haque et al., 2024)	Early clinical development
Substrate reduction therapy	Venglustat	Glucosylceramide synthase inhibition	Phase II	Reduced GlcCer and GlcSph levels in CSF in GD type 3 patients; Improved brain connectivity on fMRI (Schiffmann et al., 2023)	Potential off-target effects, long-term safety in PD
Gene therapy	AAV9-GCase (PR001)	AAV-mediated GBA1 gene delivery	Phase I/II	Restored GCase activity, reduced α-Syn burden in preclinical models; Safety and tolerability in early trials (Gegg et al., 2022; Winston et al., 2025)	Immunogenicity of AAV vectors, limited distribution, surgical delivery requirements
	AAVrh10-thermostable dGCase	Engineered thermostable GCase variant	Preclinical	Superior stability and activity, broader distribution, prolonged survival in nGD models (Milenkovic et al., 2024)	Preclinical stage
Enzyme replacement therapy	RVG- or TfR-conjugated GCase, nanoparticle ERT	Engineered BBB-penetrant GCase variants	Preclinical/investigational new drug	Improved CNS targeting and α-Syn clearance in preclinical models (Behl et al., 2021; Liang et al., 2024; Grabowski et al., 2025)	Limited BBB penetration of conventional ERT, immunogenicity

Summary of emerging therapeutic strategies for GBA1-PD, including mechanisms, clinical phases, key findings, and limitations. AAV: Adeno-associated virus; BBB: blood–brain barrier; CNS: central nervous system; CSF: cerebrospinal fluid; dGCase: deficient glucocerebrosidase; ERT: enzyme replacement therapy; fMRI: functional magnetic resonance imaging; GBA1: glucocerebrosidase 1; GCase: glucocerebrosidase; GD: Gaucher disease; GluCer: glucosylcerebroside; IL: interleukin; MAD: multiple ascending dose; nGD: neuronopathic Gaucher disease; NLRP3: NLR family pyrin domain containing 3; PBMC: peripheral blood mononuclear cell; PD: Parkinson’s disease; RVG: rabies virus glycoprotein; SAD: single ascending dose; TfR: transferrin receptor; α-Syn: α-synuclein.

### Anti-inflammatory agents

Targeting neuroinflammation is pivotal in *GBA1*-PD due to the bidirectional link between GCase deficiency and innate immune activation, which exacerbates α-syn pathology and dopaminergic neuron loss in a pathogenic feedback loop (Bo et al., 2022; Navarro-Romero et al., 2022). A major focus is inhibiting the *NLRP3* inflammasome, a key mediator of IL-1β and IL-18 release, using agents such as *MCC950*, a selective *NLRP3* inhibitor that reduces IL-1β production and motor deficits in PD murine models; it is currently in Phase II trials combined with dopaminergic therapy (Li et al., 2022). However, *MCC950* exhibits hepatotoxicity risks and off-target effects in therapeutic applications, which constrain its clinical development (Wu et al., 2024). Recent Phase II data show that MCC950 fails to improve motor scores in GBA1-PD, possibly due to hepatotoxicity and poor CNS penetration (Lanore et al., 2025). However, newer analogs, such as RRx-001 and NT-0796, which are BBB-permeable and covalently bind NLRP3, show promise in preclinical models (Haque et al., 2024). The study demonstrates that rebamipide significantly ameliorates neuroinflammation, dopaminergic neuron loss, and motor dysfunction in PD models through targeted inhibition of the *NLRP3* inflammasome (Lim et al., 2025). Complementary strategies include *LRRK2* kinase inhibitors, which normalize lysosomal defects and cytokine overproduction in *GBA1*-mutant astrocytes (Sanyal et al., 2020), and PGRN-derived therapeutics (e.g., BBB-penetrant ND7 peptide) that alleviate microgliosis/astrogliosis in mice (Zhao et al., 2023).

Neuroinflammation in *GBA1*-PD is mitigated by restoring lysosomal function and enhancing α-syn clearance. Small-molecule chaperones such as ambroxol stabilize mutant GCase, suppress M1-like microglial activation, and are under clinical evaluation in *GBA1*-PD patients (Gegg et al., 2022; Vieira and Schapira, 2022). Substrate reduction therapy (e.g., venglustat) indirectly dampens neuroinflammation by lowering GlcCer levels (Gegg et al., 2022; Vieira and Schapira, 2022). Additionally, immunomodulatory approaches include restoring GCase in microglia/ macrophages to reduce neuroinflammation in *Drosophila* (Vincow et al., 2023) and blocking IL-1β/TNF-α signaling (Kweon et al., 2024a, b). Autophagy-lysosomal pathway enhancers, such as rapamycin (an mTOR inhibitor), reduce NF-κB-driven inflammation and rescue gut-brain axis dysfunction in *GBA1*-deficient models (Atilano et al., 2024), while gene therapy (AAV9-*GBA1*, PR001) restores GCase activity and reduces α-syn burden (Gegg et al., 2022).

Key challenges include *GBA1* mutation heterogeneity, which differentially impacts enzymatic deficiency and inflammatory responses, necessitating mutation-tailored approaches (Bo et al., 2022; Kweon et al., 2024b). BBB penetrance remains critical; ND7 and ambroxol show promise due to proven BBB permeability (Zhao et al., 2023). Future efforts should prioritize combination therapies to synergistically target lysosomal dysfunction, substrate accumulation, and neuroinflammation. Biomarker-driven clinical trials validating these multi-pronged strategies in *GBA1* mutation carriers are essential.

### Gene therapy

Gene therapy offers a transformative approach for *GBA1*-PD by directly targeting the genetic defect, enabling long-lasting, disease-modifying effects with curative potential (Szunyogh et al., 2025). CRISPR-Cas9 editing corrects the *N370S* mutation in patient-derived iPSCs, restoring GCase activity and lysosomal function while facilitating disease modeling and cell replacement strategies (Yarkova et al., 2024). Adeno-associated virus (AAV) vectors deliver functional GBA1 to the brain, as evidenced by preclinical studies: Neonatal AAV-wtGBA1 administration in α-syn 3K mutant mice reduces lipid-rich α-syn aggregates, increases tetramer:monomer ratio, enhances lysosomal cathepsin maturation, and rescues motor/cognitive deficits (Glajch et al., 2021), while unilateral intracerebroventricular AAVrh10 encoding thermostable dGCase achieves bilateral GCase distribution, mitigates neuroinflammation, normalizes gene expression, and prolongs survival in neuronopathic GD models (Milenkovic et al., 2024). Clinical trials confirm the safety and tolerability of AAV-GCase in PD patients, highlighting its potential to improve neurological function (Winston et al., 2025). The immunogenicity challenges of AAV contribute to clinical response rates below 50% in certain indications, and multiple engineering and immunomodulatory strategies are being actively developed to overcome this bottleneck (Keeler et al., 2025).

Beyond viral vectors, innovative strategies address *GBA1* pathologies through optimized timing or cell-mediated delivery. Fetal gene therapy exploits BBB immaturity: intracranial adeno-associated virus vectors injection in utero achieves global CNS distribution, abolishing neurodegeneration, reducing neuroinflammation, and extending lifespan to 18 weeks in nGD mice (*versus* 15 days untreated), with ultrasound-guided delivery in non-human primates supporting translational feasibility. Neonatal intervention proved inferior, underscoring the necessity of prenatal timing to preempt irreversible damage (Massaro et al., 2018). iPSC-derived neural precursor cells provide a combined cell and gene therapy approach: intravenously administered engineered cells engraft broadly, differentiate into neural lineages, and secrete functional GCase, which reduces α-syn aggregates by 50%, elevates neurotrophic factors, and rescues mitochondrial/lysosomal dysfunction in nGD mice, suggesting applicability to *GBA1*-PD (Peng et al., 2023).

Protein engineering and vector design enhance therapeutic efficacy. Thermostable dGCase, delivered via AAVrh10, exhibits superior stability and activity compared to wild-type GCase, leading to reduced GlcCer accumulation, improved motor function, and extended survival in chronic nGD mice (Milenkovic et al., 2024). Such refinements optimize lysosomal targeting, substrate clearance, and neuronal survival (Abeliovich et al., 2021), positioning gene therapy as a precision medicine strategy that may surpass symptomatic treatments for *GBA1*-PD.

### Lifestyle interventions

Lifestyle factors significantly impact PD risk, progression, and symptom management by influencing key pathological mechanisms, including neuroinflammation, mitochondrial function, *GBA1* activity, and α-syn handling. High levels of sustained physical activity are associated with reduced PD incidence and progression. For example, cohort studies show women maintaining high daily activity had a 25% lower PD risk over decades compared to sedentary individuals, while exercise improves motor symptoms and slows neurodegeneration, potentially benefiting *GBA1* mutation carriers (Schootemeijer et al., 2020; Portugal et al., 2023). Animal models demonstrate exercise reduces microglial activation and pro-inflammatory cytokines (IL-1β, TNFα), pathways elevated in *GBA1*-PD (de Almeida et al., 2022).

Dietary quality profoundly affects PD risk. Adherence to healthy patterns such as the Mediterranean diet reduces risk by 36% (Miliukhina et al., 2020). Protective components include fiber, folate, vitamin B12, and unsaturated fats, which may suppress microglial activation and *NLRP3* inflammasome signaling (Miliukhina et al., 2020). Conversely, high intake of carbohydrates, added sugars, and dairy increases risk, potentially via oxidative stress (Janssen Daalen et al., 2022). For *GBA1*-PD specifically, reducing sphingolipid intake (e.g., limiting dairy/meat) lowers substrate burden, potentially mitigating GlcCer accumulation and neurodegeneration, as seen in zebrafish models (Bo et al., 2022; Lelieveld et al., 2022). Beverage choices also matter: coffee consumption reduces risk, with its component caffeine demonstrating dopaminergic neuroprotection (Waheeb et al., 2025), particularly enhanced in carriers of genes such as *LRRK2* (Ong et al., 2023). Moderate alcohol intake may lower PD risk, potentially due to anti-inflammatory effects, but excessive consumption can damage the BBB (Jung et al., 2023).

Other critical lifestyle factors include avoiding heavy smoking, which despite nicotine’s neuroprotective link to a ~50% lower PD risk compared to never-smokers, increases mortality and may accelerate progression (Rose et al., 2024); managing chronic stress, which exacerbates *GBA1*-PD microglial activation and ER stress (Sanyal et al., 2020), potentially counteracted by mindfulness practices (yoga, meditation) lowering inflammation (Miliukhina et al., 2020); minimizing prolonged sitting (> 9 hours/day), linked to worse cognition and PD severity (Jiao et al., 2025b); and prioritizing sleep hygiene, as disrupted sleep impairs glymphatic α-syn clearance and worsens neuroinflammation in *GBA1* models (Zhu et al., 2025). Collectively, while requiring further clinical validation, these lifestyle strategies, which target inflammation, protein handling, metabolic stress, and neuronal resilience, offer a multi-targeted approach to potentially modify PD course and complement pharmacological therapies (Vieira and Schapira, 2022; Kuppuramalingam et al., 2024).

## Conclusion

GBA1-PD represents a complex and aggressive clinical subtype characterized by early onset, rapid progression, and prominent cognitive impairment. This review has systematically synthesized the current understanding of its multifaceted pathogenesis, highlighting the critical interplay between lysosomal dysfunction, protein aggregation, and neuroinflammation. The core pathology centers on GCase deficiency, which triggers a self-reinforcing cycle of glycosphingolipid accumulation (particularly GlcCer and GlcSph) and α-syn aggregation (Granek et al., 2023; Zhang et al., 2024). This cycle is further amplified through impaired autophagy-lysosomal pathways, mitochondrial dysfunction, and robust neuroimmune activation involving microglia, astrocytes, and the gut-brain axis (Bo et al., 2022; Cossu et al., 2023; Huang et al., 2023).

The clinical heterogeneity of GBA1-PD is influenced by both genetic and epigenetic factors. Ethnicity-specific variant spectra, such as L444P in East Asians, N370S in Ashkenazi Jewish populations, and T369M in Turkish patients, together with co-occurring monogenic variants, contribute to diverse phenotypic manifestations (Zhou et al., 2023; Koros et al., 2024; Koç Yekedüz et al., 2025). Epigenetic modifications, including DNA methylation of the GBA1 promoter and dysregulation of non-coding RNAs, further refine disease penetrance and progression (Toker et al., 2021; Zhang et al., 2024). These findings highlight the importance of precision medicine strategies that integrate genetic, epigenetic, and biomarker profiles to stratify patients and tailor interventions (Orrú et al., 2025).

Significant advances have been made in therapeutic development for GBA1-PD. Pharmacological chaperones, NLRP3 inhibitors, substrate reduction therapy, and AAV-based gene therapy show promise in early studies (Haque et al., 2024; Istaiti et al., 2025). Yet challenges remain, including limited BBB penetration, mutation-specific efficacy, and viral vector immunogenicity (Keeler et al., 2025; Lanore et al., 2025). Future directions should focus on reliable biomarkers, advanced human-derived models, and combinatorial strategies to guide precision medicine approaches (Orrú et al., 2025).

Our group is actively exploring the role of GBA1 in modulating both peripheral and CNS inflammation using diverse preclinical models. Building on prior work with PINK1 and PARK2 in experimental autoimmune encephalomyelitis, which demonstrated regulation of both mitophagy and immune responses (Cossu et al., 2021, 2022, 2024), we are investigating how GBA1 dysfunction influences neuroimmune interactions. This work provides novel mechanistic insights and testable hypotheses, such as whether peripheral immune modulation can alter α-syn propagation or disease severity, and whether patient stratification by mutation type predicts therapeutic response.

Overall, GBA1 dysfunction lies at the intersection of neuroinflammation and neurodegeneration in PD. Despite persistent challenges, the integration of mechanistic insights, therapeutic innovations, and precision medicine frameworks provides unprecedented opportunities to develop effective disease-modifying therapies.

## Data Availability

*Not applicable.*
